# NFATc3 and VIP in Idiopathic Pulmonary Fibrosis and Chronic Obstructive Pulmonary Disease

**DOI:** 10.1371/journal.pone.0170606

**Published:** 2017-01-26

**Authors:** Anthony M. Szema, Edward Forsyth, Benjamin Ying, Sayyed A. Hamidi, John J. Chen, Sonya Hwang, Jonathan C. Li, Debra Sabatini Dwyer, Juan M. Ramiro-Diaz, Wieslawa Giermakowska, Laura V. Gonzalez Bosc

**Affiliations:** 1 Stony Brook University, Department of Technology and Society, College of Engineering and Applied Sciences, Stony Brook, NY, United States of America; 2 The Stony Brook Medicine SUNY at Stony Brook Internal Medicine Residency Program at John T. Mather Memorial Hospital, Port Jefferson, NY, United States of America; 3 Department of Occupational Medicine, Epidemiology, and Preventive Medicine, Hofstra Northwell School of Medicine at Hofstra University, Hempstead and Manhasset, NY, United States of America; 4 Three Village Allergy & Asthma, PLLC, South Setauket, NY, United States of America; 5 Columbia University Child Psychiatric Epidemiology Group, New York, NY, United States of America; 6 Stony Brook University School of Medicine M.D. with Scholarly Recognition Program, Stony Brook, NY, United States of America; 7 Department of Internal Medicine, Bronx Veterans Affairs Medical Center Internal Medicine Residency Program, Bronx, NY, United States of America; 8 Biostatistics and Data Management Core, John A. Burns School of Medicine, University of Hawaii, Honolulu, Hawaii, United States of America; 9 Department of Pathology, SUNY Stony Brook School of Medicine, Stony Brook, NY, United States of America; 10 Department of Cell Biology and Physiology, University of New Mexico Health Sciences Center, Albuquerque, NM, United States of America; University of Illinois at Chicago College of Medicine, UNITED STATES

## Abstract

Idiopathic pulmonary fibrosis (IPF) and chronic obstructive pulmonary disease (COPD) are both debilitating lung diseases which can lead to hypoxemia and pulmonary hypertension (PH). Nuclear Factor of Activated T-cells (NFAT) is a transcription factor implicated in the etiology of vascular remodeling in hypoxic PH. We have previously shown that mice lacking the ability to generate Vasoactive Intestinal Peptide (VIP) develop spontaneous PH, pulmonary arterial remodeling and lung inflammation. Inhibition of NFAT attenuated PH in these mice suggesting a connection between NFAT and VIP. To test the hypotheses that: 1) VIP inhibits NFAT isoform c3 (NFATc3) activity in pulmonary vascular smooth muscle cells; 2) lung NFATc3 activation is associated with disease severity in IPF and COPD patients, and 3) VIP and NFATc3 expression correlate in lung tissue from IPF and COPD patients. NFAT activity was determined in isolated pulmonary arteries from NFAT-luciferase reporter mice. The % of nuclei with NFAT nuclear accumulation was determined in primary human pulmonary artery smooth muscle cell (PASMC) cultures; in lung airway epithelia and smooth muscle and pulmonary endothelia and smooth muscle from IPF and COPD patients; and in PASMC from mouse lung sections by fluorescence microscopy. Both NFAT and VIP mRNA levels were measured in lungs from IPF and COPD patients. Empirical strategies applied to test hypotheses regarding VIP, NFATc3 expression and activity, and disease type and severity. This study shows a significant negative correlation between NFAT isoform c3 protein expression levels in PASMC, activity of NFATc3 in pulmonary endothelial cells, expression and activity of NFATc3 in bronchial epithelial cells and lung function in IPF patients, supporting the concept that NFATc3 is activated in the early stages of IPF. We further show that there is a significant positive correlation between NFATc3 mRNA expression and VIP RNA expression only in lungs from IPF patients. In addition, we found that VIP inhibits NFAT nuclear translocation in primary human pulmonary artery smooth muscle cells (PASMC). Early activation of NFATc3 in IPF patients may contribute to disease progression and the increase in VIP expression could be a protective compensatory mechanism.

## Introduction

Pulmonary hypertension (PH) is an important clinical indicator of the severity of both IPF and COPD [1]. In patients with IPF, idiopathic PH (IPAH), and COPD, pulmonary vascular remodeling may lead to pulmonary hypertension and cor pulmonale [2].

Nuclear factor of activated T cells (NFAT) belongs to a family of four isoforms of Ca^2+^/calcineurin-dependent transcription factors which play an important role in immune function [3]. Besides the importance of this family of transcription factors in immune regulation, the isoforms NFATc3 and NFATc2 have been particularly implicated in the development of PH [4–7]. NFATc3 is linked to pulmonary arterial smooth muscle (PASMC) hyperplasia and hypertrophy in chronic hypoxia-induced PH [4,6,7]. In addition, it has been shown that NFATc3 represses the expression of voltage-dependent potassium channels (Kv 2.1) [8] and large conductance potassium channel β subunit [9], upregulates the expression of transient receptor potential cation channel subtype C1 (TRPC1) [10] and smooth muscle α-actin [6,11], suggesting it might be implicated in the regulation of vascular smooth muscle contractility. Furthermore, NFATc2 is implicated in the downregulation of Kv1.5 expression, membrane depolarization, proliferation and resistance to apoptosis of PASMC in idiopathic PAH patients and in rats with monocrotaline-induced PH [5,12]. However, little is known about the role of NFAT in pulmonary vascular endothelium. Recent reports show that vascular endothelial growth factor (VEGF)-mediated activation of calcineurin/NFAT signaling upregulates angiopoietin-2 in lung endothelial cells [13]. In the systemic circulation, it has been shown that NFAT activation regulates angiogenesis [14,15]. A delicate balance of angiogenic and angiostatic factors regulates vessel homeostasis in normal physiologic conditions in the lungs. There is increased angiogenesis in COPD [16]. However, controversy exists regarding whether angiogenesis is increased or decreased in IPF [17]. Therefore, NFAT could play different roles depending on the vascular cell in which it is activated and the underlying clinical disease process.

We have shown previously that Vasoactive Intestinal Peptide (VIP) knockout mice develop spontaneous pulmonary hypertension and T lymphocytic infiltration of the airways and vasculature [18,19] and that administration of VIVIT peptide (an inhibitor of NFAT) reduces inflammation and pulmonary arterial remodeling in these mice. In addition, VIP attenuates monocrotaline-induced pulmonary hypertension in rats [20] in which NFAT has been implicated in disease pathogenesis. These studies then suggest a link between VIP and NFAT. VIP has been described, with respect to the pulmonary circulation, merely as a vasodilator of pulmonary vessels [21–25] but also suppresses vascular smooth muscle cell proliferation [26]. These actions are mediated primarily by reducing intracellular Ca^2+^ via activation of the cAMP/protein kinase A (PKA) pathway [26].

These findings led us to test the following hypotheses:

1) VIP reduces NFATc3 transcriptional activity. 2) Lung NFATc3 activation and VIP mRNA expression are associated with disease severity in individuals with IPF and COPD.

## Materials and Methods

### Animals

All protocols employed in this study were reviewed and approved by the Institutional Animal Care and Use Committee of the University of New Mexico, School of Medicine (Albuquerque, NM).

Adult (2–3 month old) male 9x-NFAT-luciferase reporter (NFAT-luc) mice (20–25 g) were used. NFAT-luc mice were provided by Dr. Jeffery D. Molkentin (Department of Pediatrics, Children’s Hospital Medical Center, Cincinnati, Ohio) [27]. These mice carry 9 copies of an NFAT binding site from the IL-4 promoter (5′-TGGAAAATT-3′) positioned 5′ to a minimal promoter from the α-myosin heavy chain gene (−164 to +16) and inserted upstream of the luciferase reporter [27]. VIP knockout (KO) mice, backcrossed to C57BL/6 mice, were obtained from Dr. Colwell, Mental Retardation Res. Ctr., Univ. of California, Los Angeles [28]. All mice were euthanized by removing the heart and lungs under pentobarbital anesthesia.

#### Chronic hypoxia exposure

Animals designated for exposure to chronic hypobaric hypoxia (CH) were housed in a hypobaric chamber with barometric pressure maintained at ~380 Torr for 2 days. Control animals were housed at ambient barometric pressure (normoxia, N, ~630 Torr). All animals were maintained on a 12:12-h light-dark cycle.

#### Animal treatments

Treatment was started one day prior to CH exposure, followed by 2 days during CH. NFAT-luc mice were treated with 0.166 mg/kg/day VIP administered s.c. via osmotic pumps (Alzet). Control animals received vehicle alone (saline).

### Human samples

Studies were authorized by a SUNY Stony Brook and UNM HSC Institutional Review exempt materials transfer agreement. RNA-later protected and formalin fixed-paraffin embedded lung tissue, with associated de-identified clinical data from patients with COPD or IPF, were obtained from the NIH Lung Tissue Research Consortium (LTRC).

Clinical and demographic data (S1 Table) were de-identified and anonymously-coded by the NIH and divided in the following groups: group 1 (control): smokers with slightly compromised lung function (FEV1 > = 80%), group 2: smokers with mildly compromised lung function (FEV1 is 50–80%); group 3: smokers with severely compromised lung function (FEV1 <50%) and group 4: smokers with IPF (FVC < 50%). Each group consisted of 9–10 patients.

### Assays

#### Luciferase activity

Intrapulmonary arteries were isolated from NFAT-luc mice and lysed (Promega buffer). Luciferase activity was measured using a Luciferase Assay System kit (Promega, Madison, WI), and light detected with a luminometer (TD20/20, Turner). Protein content was determined by the Bradford method (BioRad, Hercules, CA) and used to normalize luciferase activity.

#### Cell culture and NFATc3 nuclear import

Human PASMC (Life Technologies, Carlsbad, CA) were grown in poly-L-lysine (10 μg/ml) coated flasks in Growth Medium 231 (Life Technologies, Carlsbad, CA) at 37°C in 5% CO_2_ with controlled humidity. Cells were electroporated (Nucleofector, Lonza, Basel, Switzerland) with NFATc3-EGFP (enhanced green fluorescent protein) expression vector. This vector was created by Dr. F. McKeon (Harvard University, Cambridge, MA) and kindly provided by Dr. L.F. Santana (Washington State University, Seattle, WA). Electroporated cells were seeded on microscope coverslips coated with poly-L-lysine, cultured in Growth Medium 231 (Life Technologies, Carlsbad, CA) at 37°C in 5% CO_2_ with controlled humidity. Before any experiment, cells were cultured for at least 48 hrs. in differentiation media that contained 1% FBS and 30 μg/ml heparin (Life Technologies, Carlsbad, CA). In all the experiments, cells were pre-incubated with a myosin light chain kinase peptide inhibitor (MLCK, 1 μM) to prevent cell contraction and detachment. Then, cells were subjected to different treatments described in results section. Nuclear EGFP fluorescence was monitored using a NIKON Diaphot 300 at 200X magnification. Individual cells were imaged once every minute for 30 minutes. Images were captured using Andor IQ 1.9 software (Belfast, UK). Nuclear fluorescence (F) was background corrected and expressed as fold change from baseline nuclear fluorescence (F/F_0_) (Metamorph Universal Imaging software, Molecular Devices, Sunnyvale, CA).

#### NFATc3 immunofluorescence confocal microscopy

Paraffin lung sections from WT and VIP KO mice were de-paraffinazed, subject to antigen retrieval process, permeabilized, blocked for nonspecific binding, and primary antibodies [rabbit polyclonal anti-NFATc3; 1:100 (Santa Cruz Biotechnology) and anti-α-actin; 1:250 (Sigma)] were prepared in 0.2% gelatin in PBS and applied overnight at 4°C. Secondary antibodies [anti-rabbit Cy5 and anti-mouse Cy3; 1:500 (Jackson ImmunoResearch Laboratories)] were prepared in 0.2% gelatin in PBS and applied for 1 h at room temperature. Nuclei were stained using SYTOX green (1:5,000 in PBS; Molecular Probes). Sections were examined using a x63 objective on Leica TCS SP5 laser scanning confocal microscope. Specificity of immune staining was confirmed by the absence of fluorescence in tissues incubated with primary or secondary antibodies alone. For scoring of NFATc3-positive nuclei, multiple fields for each vessel were imaged and counted by two independent observers blinded to the treatment using MetaMorph software (Universal Imaging) as previously described [6,29,30]. The software was programmed so that individual pixels would appear white instead of yellow if the green nucleic acid stain and red NFATc3 stain colocalized. Thus a cell was considered positive if co-localization (white) was uniformly distributed in the nucleus and negative if no co-localization (green only) was observed.

Paraffin lung sections from the four patient’s groups were processed and immuno-stained as described above. NFATc3 integrated intensity (AU) was measured in the threshold individual channels in pulmonary arterial smooth muscle and endothelial cells and in airway smooth muscle and epithelial cells using Metamorph software. The same threshold was applied to every image.

#### Airway and vascular remodeling

Paraffin lung sections from the four patient’s groups were de-paraffinazed and stained with hematoxylin and eosin for standard morphologic analysis.

### Statistical analysis and models

#### Animal testing

Animal data were expressed as mean ± SEM. Statistical significance was tested at 95% (p<0.05) confidence level using unpaired *t*-test or one-way ANOVA followed by Newman-Keuls multiple comparisons test or Kruskal-Wallis One Way Analysis of Variance on Ranks if data did not pass the equal variance test.

#### Human testing

A variety of tests were conducted on the human data to tease out robust relationships in our hypothesis-testing. Using the data from the human samples across the four disease groups we perform multivariate analyses of FEV1/FVC% and remodeling outcomes against our key independent variables NFATc3 fluorescence intensity in airway and pulmonary vascular cells as well as the percent NFATc3 positive nuclei. Other independent variables of interest included were VIP and NFATc3 mRNA levels. Gender, age, race, and smoking intensity were the confounding factors included in the analysis.

#### Measurement of independent variables

Given the small sample sizes in the remodeling analysis, factor analysis was used to retain only the common factors NFATc3 intensity for airway epithelial and smooth muscle cells and for pulmonary endothelial and smooth muscle cells. Factor analysis is a data reductionist methodology that combines multiple indicators of latent variables in order to capture the commonalities and minimize noise. Two types of factor analysis were used. Exploratory factor analysis of a correlation matrix in order to capture the shared variances in the four indicators was used first. The prior was that the two airway cells could be collapsed into one latent factor and the two pulmonary cells could do the same. The exploratory factor analysis results confirmed our priors in that the first two eigenvalues explained most of the variation in the four indicators. The first factor loads predominantly on the NFATc3 airway intensity scores while the second factors loads onto the pulmonary vascular intensity scores. Confirmatory factor analysis was also run but since the exploratory is less restrictive and the assumption of two factors did not need to be imposed, the exploratory results were used. Factor analysis results are reported in Appendix A. These analyses confirmed our hypotheses that the indicators can be matched to two mutually exclusive domains related to airway and pulmonary vascular NFATc3 staining intensities. These constructed indices were only used in the remodeling outcomes given smaller samples with less power. For the models of FEV1/FVC %, the original variables were used.

Our hypothesis is that the effects of the NFATc3 activation vary by disease type and severity. In other words, the interactive effects of the key variables of interest with disease type/severity were captured in the statistical analysis. To do this, a predicted probability of disease type and severity (IPF and COPD stage 3) allowing for an interaction between the NFATc3 expression and nuclear translocation indicators and disease was constructed. The intensity scores were included separately to test for an independent effect on FEV/1FVC % and their impact nested with disease type.

## Results

### VIP regulates NFATc3 activity

**Fig 1:**
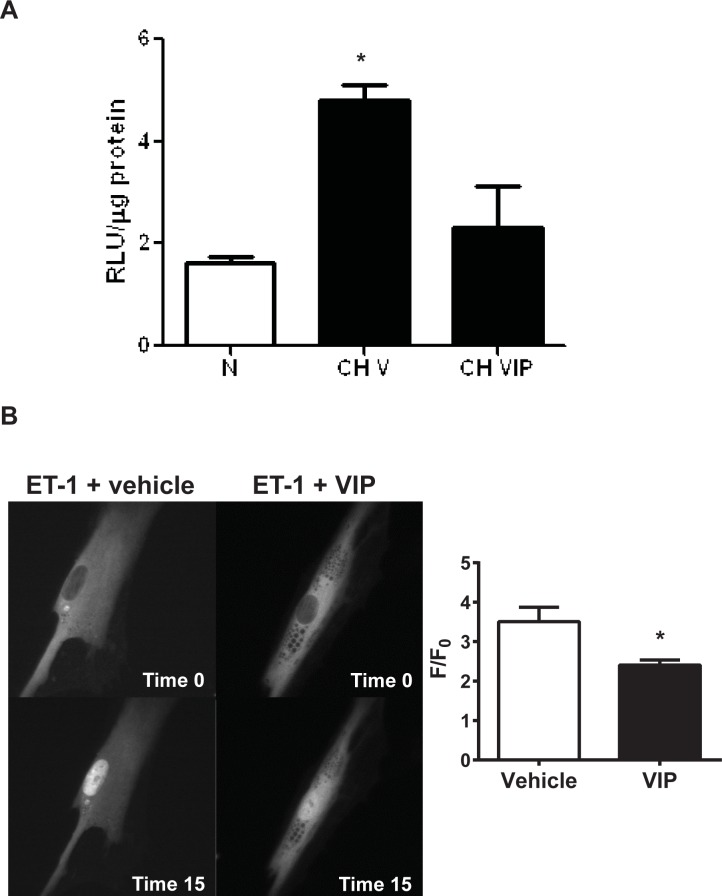
VIP attenuates NFAT activation. A) VIP administration inhibited CH-induced NFAT activation in isolated mouse intrapulmonary arteries. RLU = relative luciferase units. N = normoxia, CH V = 2 days chronic hypoxia. CH VIP = VIP (0.166 mg/kg/day) in osmotic pump one day prior and during CH exposure for 2 days. *p<0.05 vs N and CH VIP, n = 3–5 mice. ANOVA followed by Newman-Keuls. B) VIP attenuated ET-1-induced NFATc3-EGFP nuclear import in human PASMC. *p<0.05, n = 5 cells. t-test.

We have previously demonstrated that 2 days of CH exposure causes NFATc3 activation in isolated intra pulmonary arteries in mice [6]. In the same study, we have shown that NFATc3 is the only isoform activated by CH and that its activation is associated with increased NFATc3 nuclear localization in PASMC [6]. Here, we show that CH-induced NFAT activation in isolated intra pulmonary arteries was abrogated by treating mice with VIP one day prior and during CH exposure (Fig 1A).

**Fig 2:**
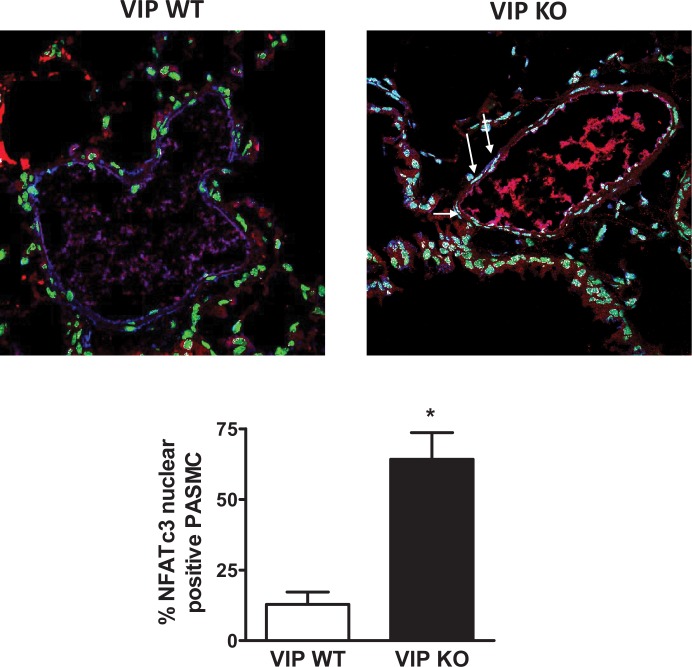
PASMC NFATc3 nuclear accumulation is enhanced in VIP KO mice. Top: representative immunofluorescence confocal microscopy images. Nuclei depicted in green (SYTOX), NFATc3 in red and α-actin in blue. NFATc3 nuclear co-localization in white (see white arrows). Bottom: summary of percent of PASMC with NFATc3 in the nucleus. n = 15 to 28 arteries from 6 mice each group. *p<0.001

Furthermore, VIP (1 **μ**M) attenuated ET-1 (100 nM)-induced NFATc3 nuclear translocation in cultured human PASMC (Fig 1B). Consistent with these findings, NFATc3 nuclear localization was enhanced in PASMC from VIP KO compared to WT mice (Fig 2).

Taken together these results suggest that VIP is an endogenous inhibitor of NFATc3. These findings provided the rationale for determining NFATc3 activity status in COPD and IPF patients.

### Airway and vascular remodeling in COPD and IPF patients

**Fig 3:**
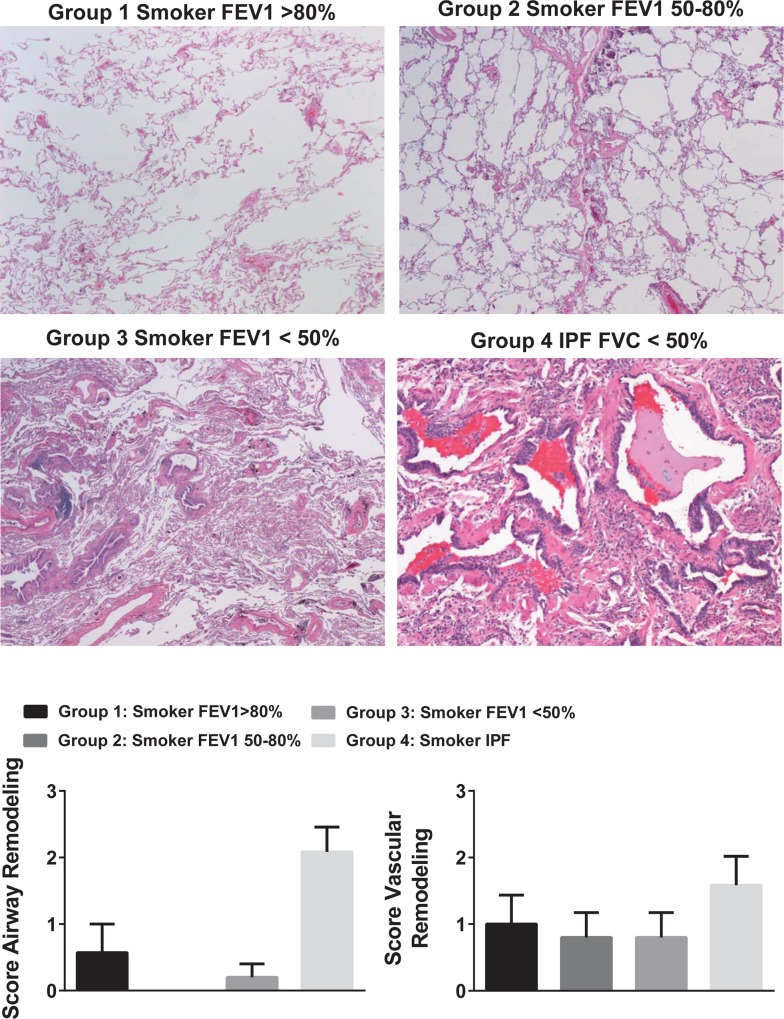
Airway and vascular remodeling graphed based on severity of histology. Group 1 COPD FEV1<50%; Group 2 COPD FEV 50–80%; Group 3 COPD, control, FEV1>80%; Group 4 IPF FVC<50%. One-way analysis of variance (ANOVA) determined that means of airway remodeling scores were significantly different (*p*<0.002), and *Post hoc* Tukey’s Multiple Comparison Test found that Group 4 compared to each of the other three groups was significant for differences in airway remodeling (*p*<0.05 for each comparison). n = 5–7

Grading of histology by a pathologist blinded to identities of the samples showed significant airway remodeling in the IPF group (group 4) compared to the other three groups (Fig 3). There was no significant difference in vascular remodeling among the four groups (Fig 3).

### NFATc3 activity and expression in lungs from COPD and IPF patients–Descriptive Outcomes

**Fig 4:**
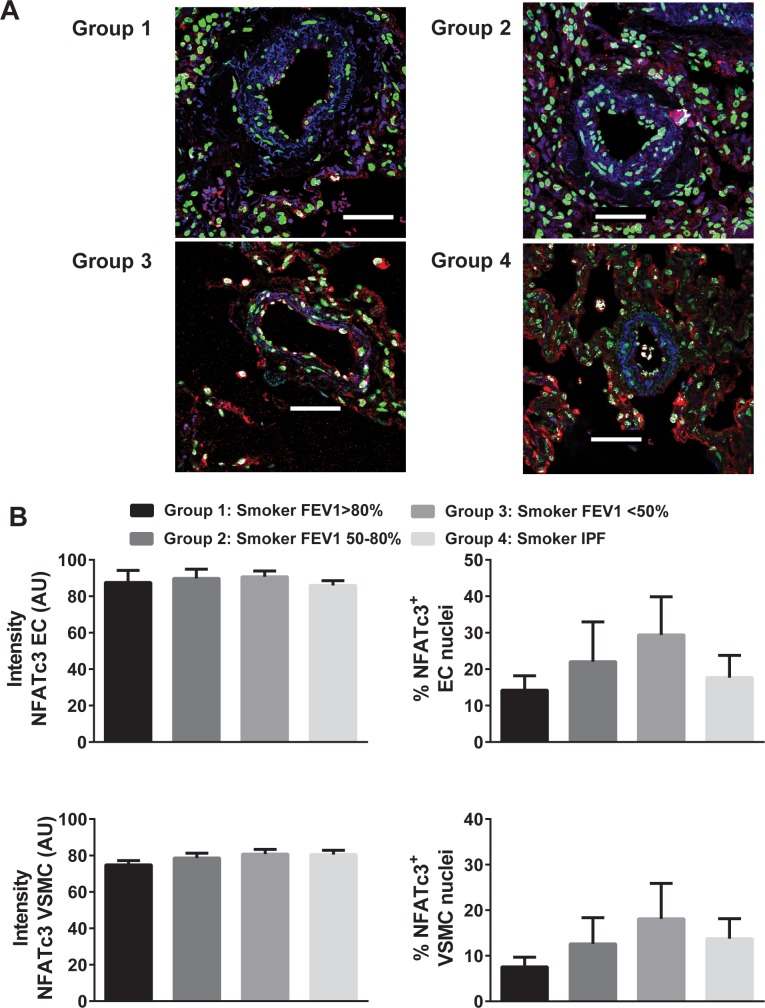
NFATc3 activity and expression in pulmonary artery endothelial and smooth muscle cells from lungs from COPD and IPF patients. A) Representative images of NFATc3 (red) and smooth muscle alpha-actin (blue) immunostaining of lung sections of patients from groups 1–4. Nuclei were stained with Sytox green. NFATc3 nuclear co-localization is shown in white. Scale bar = 50 **μ**m. B) Summary results of endothelial cell (EC) NFATc3 staining intensity, % NFATc3+ EC nuclei, vascular smooth muscle (VSMC) NFATc3 staining intensity and % NFATc3+ VSMC nuclei. N = 7–14 patients. ANOVA followed by Newman-Keuls.

**Fig 5:**
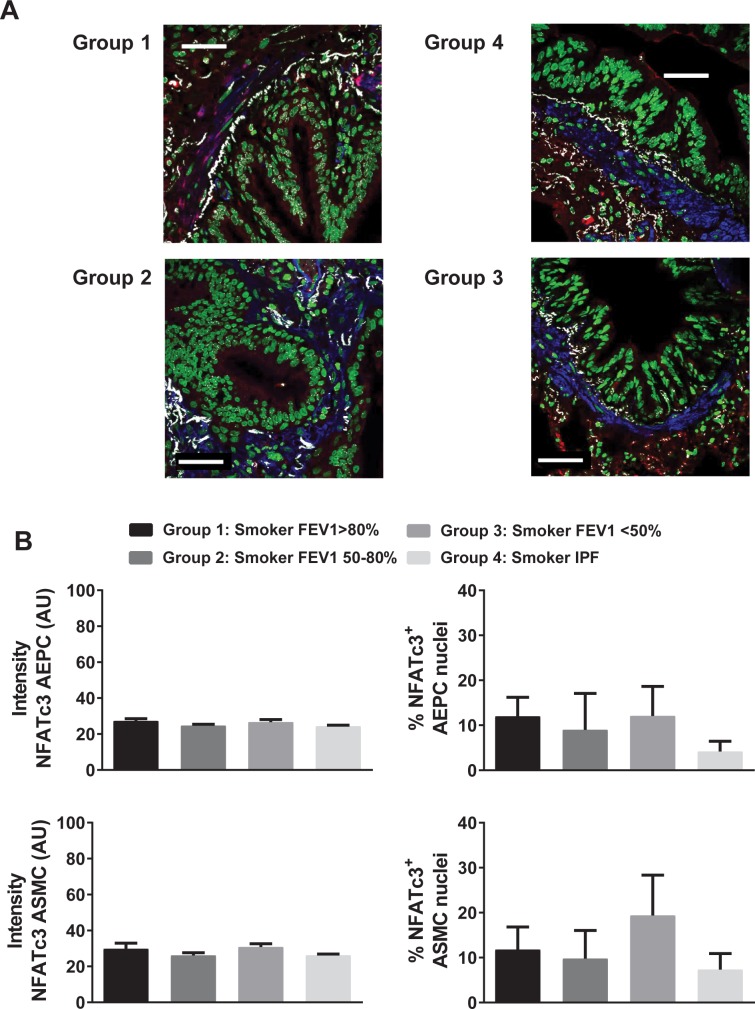
NFATc3 activity and expression in airway epithelial and smooth muscle cells from lungs from COPD and IPF patients. A) Representative images of NFATc3 (red) and smooth muscle alpha-actin (blue) immunostaining of lung sections of patients from groups 1–4. Nuclei were stained with Sytox green. NFATc3 nuclear co-localization is shown in white. Scale bar = 50 **μ**m. B) Summary results of airway epithelial cells (AEPC) NFATc3 staining intensity, % NFATc3+ AEPC nuclei, airway smooth muscle (ASMC) NFATc3 staining intensity and % NFATc3+ ASMC nuclei. N = 5–7 patients. ANOVA followed by Newman-Keuls.

It is known that NFATc3 nuclear accumulation is a primary step in the NFATc3 activation pathway. We focused on the NFATc3 isoform because it is activated in pulmonary arteries by CH [6,30] and also because VIP attenuates its activation (Fig 1). Here, the % of nuclei of PASMC, pulmonary artery endothelial cells (PAEC), airway epithelial (AEPC) and smooth muscle cells (ASMC) that were positive for NFATc3 in lung sections from the four groups of patients was determined as a marker of NFATc3 activation. The average intensity of NFATc3 staining in the same cell types was measured to determine the differences in NFATc3 protein expression among the groups. Furthermore, NFATc3 mRNA levels were assessed by real time PCR in lungs from the four groups of patients. There was no significant difference in the % of NFATc3 positive nuclei or intensity among the different groups in any of the cell types analyzed (Figs 4 and 5).

**Fig 6:**
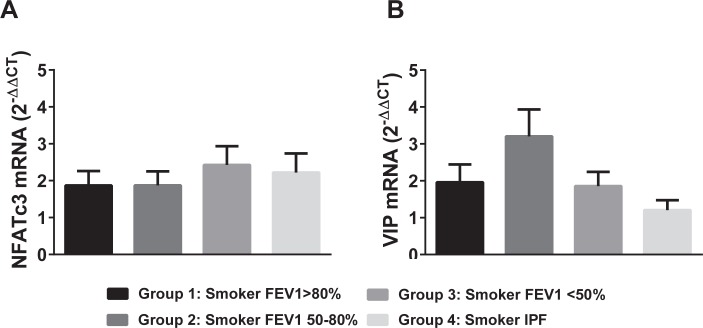
NFATc3 and VIP mRNA levels in lungs from COPD and IPF patients. NFATc3 and VIP mRNA levels were determined by real time PCR in lungs from patients from groups 1–4.

Neither was there a significant difference in NFATc3 mRNA levels among the groups (Fig 6).

Table 1 summarizes means and frequencies of the variables used in our hypotheses tests by disease type and severity.

**Table 1:** Means and Frequencies of FEV1/FVC % and Key Explanatory Variables By Disease State.

Variables	COPD Group 1 N = 14	COPD Group 2 N = 8	COPD Group 3 N = 7	IPF Group 4 N = 10
**Dependent Variables**				
FEV1/FVC %	0.97**	0.67**	0.36**	1.03**
**Independent Variables**				
%NFATc3^+^ASMC nuclei^1^	4.70/10.96	6.12/9.80	19.37	2.88/4.79
%NFATc3^+^AEPC nuclei^1^	5.34/12.48	5.61/8.98	12.08	2.80/4.66
%NFATc3^+^ PASMC nuclei	7.33	12.62	18.11	9.79
%NFATc3^+^ PAEC nuclei	13.65	22.08	29.41	15.08
% NFATc3^+^ASMC nuclei	-0.23	0.13	0.48	-0.11
% NFATc3^+^ AEPC nuclei	-0.17	-0.04	0.71	-0.24
Lung VIP mRNA	1.59	3.21	1.86	1.3
Lung NFATc3 mRNA	1.81	1.87	2.43	2.4
PASMC NFATc3 Intensity	74.85**	78.65**	80.78**	80.53**
PAEC NFATc3 Intensity	87.53**	89.81**	90.74**	85.93**
ASMC NFATc3 Intensity	29.79**	26.10**	30.82**	26.24**
AEPC NFATc3 Intensity	27.25**	24.71**	26.65**	24.39**
% White	100**	100**	85.71**	90.00**
% Female	42.9	50	71.43	40
Age	71.43**	62.75**	54.43**	49.9**
Packs Smoked/year	29.43	24.63	29.71	6.8

FEV1 (Forced Expiratory Volume 1), FVC (Forced Vital Capacity).^1^There was roughly a 40% missing value rate on the ASMC and AEPC indicators for all but Group 3. We report the means with and without the missing. We coded the missing as 0 which is why including them yields a much lower mean.

**Significant at the 5% level.

The largest and statistically significant differences were between advanced COPD (group 3) and IPF (group 4). In fact, as COPD progresses the FEV1/FVC % declined while in IPF patients it remained high. Non-whites and males were disproportionately represented in the severe COPD group. IPF patients were significantly younger than COPD patients. IPF patients smoke significantly less than COPD patients. These results supported a multivariate analysis to capture isolated effects of key independent variables.

### NFATc3 activity and expression in lungs from COPD and IPF patients–Multivariate Analysis

Table 2 reports the multivariate analysis results for predicting FEV1/FVC %.

**Table 2:** FEV1/FVC by Intensity of NFATc3 and RNA.

Variable	Model 1	Model 2	Model 3
**Airway Cell NFATc3 Intensity**			
ASMC	0.02 (0.02)	0.02 (0.01)**	0.02 *0.01)**
AEPC	-0.04 (0.04)	0.01 (0.01)	0.01 (0.01)
**Pulmonary Vascular NFATc3 Intensity**			
PASMC	0.05 (0.04)	-0.008 (0.01)	-0.03 (0.01)**
PAEC	-0.02 (0.01)**	0.0004 (0.00)	0.01 (0.003)*
%NFATc3^+^ ASMC nuclei	0.01 (0.01)	-0.01 (0.00)**	-0.01 (0.002)**
%NFATc3^+^ AEPC nuclei	-0.01 (0.01)	0.01 (0.00)*	0.01 (0.003)**
%NFATc3^+^ PASMC nuclei	-0.04 (0.03)	0.01 (0.01)	0.01 (0.01)
%NFATc3^+^ PAEC nuclei	0.02 (0.02)	0.004 (0.01)	0.01 (0.01)*
VIP mRNA	-0.13 (0.06)**	-0.07 (0.03)**	-0.08 (0.03)**
NFATc3 mRNA	-0.08 (0.05)*	0.01 (0.02)	0.03 (0.03)
NFAT Int →IPF	-	0.62 (0.14)**	0.61 (0.14)**
NFAT Int→COPD3	-	-0.64 (0.09)**	-0.64 (0.09)**
Packs per year	-0.02 (0.01)*	0.003 (0.004)	0.003 (0.004)
Female	-0.11 (0.20)	-0.21 (0.07)**	-0.20 (0.07)**
Age	-0.007 (0.006)	0.005 (0.003)	0.004 (0.003)
R-Square	0.5789	0.9684	0.9674

Model 1 does not include the impact of the NFATc3 intensities or the % positive nuclei indicators through the patient’s disease type. Model 2 allows NFATc3 intensities to work through disease type. Model 3 allows the NFATc3 intensities and % positive nuclei to interact with disease type.

**Significant at the 5% level. Standard Errors in (). n = 23.

In all models, the isolated impact of a high NFATc3 intensity in airway cells is positive on the lung functioning score and that of the pulmonary vascular NFATc3 intensity score is not statistically significant. The interesting finding reported in Table 3 is that among IPF patients with high NFAT score (NFATc3 intensities and % positive nuclei), the FEV1/FVC % is between 61% higher than among COPD patients. The score is 64% lower for COPD patients of group 3 relative to the less severe COPD patients (Group 1). Models that do not allow for the interaction between disease and NFATc3 indicators are noisier with poor model fit. This further re-enforced the differences in NFATc3 expression and nuclear translocation between IPF and COPD patients.

**Table 3:** Remodeling Outcomes Using All Intensity Indicators Working Through Disease.

Variable	Airway 1	Airway 2	Vascular 1	Vascular 2
Airway Cell NFATc3 Intensity	-0.45 (0.63)	0.81 (0.68)	-3.96 (3.01)	-2.57 (1.63)*
Pulmonary Vascular NFATc3 Intensity	0.33 (0.36)	-0.41 (0.37)	1.34 (1.76)	0.88 (0.90)
%NFATc3^+^ ASMC nuclei	-0.08 (0.02)**	-0.16 (0.06)	0.08 (0.1)	0.002 (0.15)
%NFATc3^+^ AEPC nuclei	0.06 (0.11)	-0.04 (0.16)	0.59 (0.5)	0.44 (0.38)
%NFATc3^+^ PASMC nuclei	-0.01 (0.11)	0.21 (0.15)	-0.59 (0.6)	-0.32 (0.37)
%NFATc3^+^ PAEC nuclei	0.01 (0.03)	-0.01 (0.05)	0.06 (0.2)	0.01 (0.12)
VIP mRNA	0.46 (0.20)**	0.12 (0.26)	1.32 (0.98)	1.04 (0.63)*
NFATc3 mRNA	1.52 (0.30)**	0.98 (0.44)**	1.13 (1.44)	0.75 (1.06)
NFAT Int →IPF	-4.12 (1.10)**	-2.71 (1.14)**	-4.68 (5.3)	-3.10 (2.74)
NFAT Int→COPD3	-4.70 (0.5)**	-4.39 (1.07)**	-2.00 (2.6)	-2.24 (2.58)
Packs per year	0.04 (0.03)	-0.02 (0.04)	0.16 (0.14)	0.11 (0.09)
Female	1.86 (0.71)**	2.70 (1.19)**	-2.15 (3.5)	-0.95 (2.88)
Age	-0.10 (0.02)**	-0.03 (0.02)	-0.17 (0.11)	-0.11 (0.05)**
R-Square	0.942	0.9841	0.2972	0.8841

Airway and Vascular 1 Models allow NFAT intensities alone to interact with disease type. Airway and Vascular 2 Models allow both NFAT intensities and %+ nuclei to interact with disease type.

**Significant at the 5% level. Standard Errors in (). n = 15

After controlling for confounding factors VIP mRNA levels are an important predictor of FEV1/FVC %. Those with high levels of VIP mRNA have significantly lower FEV1/FVC %. This is also true for females. Descriptive analysis was not able to capture significant differences in lung VIP mRNA levels among the groups of patients (Fig 6B). Multivariate analysis allows identification of isolated effects removing the noise from the confounding factors.

#### Remodeling results

Given models with the interaction between NFATc3 indicators and disease are the preferred specification for testing hypotheses of interest, we only report those results in Table 3 for the remodeling outcome.

#### Airway remodeling findings

Little independent impact of any of the NFATc3 indicators on airway remodeling was observed (the signs shift across the two models and are not significant). We do see a significant and negative impact of higher percent positive NFATc3 ASMC nuclei scores on the airway remodeling score (by 8%). Because of collinearity with the interaction controls this effect goes away in Model 2 because it is picked up in the interacted indicators. The main differences of the impact of higher levels of NFATc3 on airway remodeling scores work through disease with both IPF and severe COPD patients exhibiting lower remodeling outcomes relative to less severely ill COPD patients. IPF patients with high scores on the NFATc3 indicators have airway-remodeling scores that are 2.71 points lower on average compared to groups 1 and 2 COPD patients. COPD3 patients have airway-remodeling scores that are 4.39 percent lower than their less severe counterparts.

#### Vascular remodeling results

Airway cell NFATc3 expression intensity has a significant negative relationship with vascular remodeling and the effect appears to be independent of disease type for this outcome. Age and VIP mRNA levels proved to be important predictors as well.

## Discussion

Our study shows a novel link between the pro-inflammatory and pro-vascular remodeling gene NFAT, the anti-inflammatory anti-vascular remodeling gene VIP, with the diseases COPD and IPF.

IPF is a chronic fibrosing interstitial lung disease that is characterized by the histopathological pattern of usual interstitial pneumonia [31]. While the pathogenesis of IPF is largely unknown, it is characterized by irreversible fibrosis and bronchiolar honeycombing, and involves genetic abnormalities [32]. Accumulation of fibroblasts, myofibroblasts and extracellular matrix proteins leads to hypoxia within the lung [32]. Hypoxia causes fibroblast [33] and pulmonary arterial smooth muscle proliferation [4] in an NFAT-dependent manner. On the other hand, it has been shown that hypoxia decreases lung VIP levels in rats [34] and VIP can cause relaxation and inhibition of vascular smooth muscle cell proliferation [26]. The property of VIP to inhibit NFATc3 in mouse pulmonary artery smooth muscle both *in vivo* and in culture, and the finding that NFAT activity is enhanced *in vivo* in pulmonary artery smooth muscle from VIP KO mice, strongly supports the concept that VIP and NFAT are inextricably intertwined. VIP appears to act as an endogenous inhibitor of NFAT activity, attenuating pulmonary hypertension. Our determination that NFAT activity in IPF patient lungs is associated with higher FEV1/FVC ratio than that of COPD patients in the earlier stages of the disease suggests activation occurs early in the throes of IPF pathogenesis. NFATc3 expression and activity negatively correlates with airway remodeling in IPF patients but no significant correlation was observed with vascular remodeling. We further show that there is a significant positive correlation between NFATc3 mRNA expression and VIP mRNA expression only in lungs from IPF patients. These results suggest that downstream consequences of lung fibrosis leading to hypoxia and pulmonary hypertension can conceivably be due to a dysregulation in the balance between NFAT activity and VIP levels.

COPD frequently causes pulmonary hypertension because is a disease characterized by chronic airflow limitation resulting and hypoxemia from an abnormal inflammatory response to gas and particle inhalation, and is largely irreversible and progressive [35]. COPD involves alveolar emphysematous dilation and bronchiolar inflammation with a predominance of CD8^+^ T lymphocytes and neutrophils [36]. It has been recently shown that the number of CD8^+^/NFATc2^+^ T cells is increases in the lungs of COPD patients compared to controls and that NFAT inhibition with either cyclosporine or calcium-release activation calcium channels inhibitor (syntax-66) produces greater anti-inflammatory effects on COPD CD8^+^ cells than corticosteroids [37]. Furthermore, NFAT is up-regulated in peripheral blood mononuclear cells of subjects with severe COPD [36]. Consistent a possible role of NFAT in the pathogenesis of the inflammation present in COPD, we have previously demonstrated that NFATc3 is required for CH-induced pulmonary hypertension in mice [4,6]. Furthermore, silencing of stromal interaction molecule 1 (STIM1), a Ca^+2^ sensor on the endoplasmic reticulum, attenuated hypoxia-induced increases in rat pulmonary artery smooth muscle cell proliferation by inhibiting NFATc3 nuclear import [38] further supporting a role for NFATc3 in CH-induced pulmonary hypertension in rats. Contrary to our expectations, we found no significant differences in NFATc3 expression and/or activity among patients with different degrees of severity of COPD in any of the lung cells in which it was determined. When a multivariate analysis was used, NFATc3 expression/activity scores were 64% lower for COPD patients of group 3 relative to the less severe COPD patients (Group 1) and VIP mRNA lung levels were higher in those COPD patients with lower FEV/FVC ratios. These results suggest that VIP is upregulated in the lung of severe COPD patients and might be inhibiting NFAT.

Since NFAT activity is higher in IPF with higher FEV1/FVC ratios compared to COPD patients, indicating a late-phase disease state, it is plausible that treating smokers with VIP would reduce the risk of being diagnosed with IPF and that treating early IPF with VIP may inhibit progression of airway and vascular remodeling. In support of this possibility, VIP has been successfully used to improve symptoms and function in COPD patients, supporting its role in counteracting NFAT [39]

In conclusion, NFATc3 and VIP inextricably linked via a regulatory relationship in both COPD and IPF, supporting the concept that pathogenesis is mediated by NFATc3 and VIP may have a novel therapeutic role.

## Supporting Information

S1 Table Clinical and Demographic Data. Standard Errors in (). Gender: 1-Male, 2-Female. Race: 1-White (Caucasian), 2-Hispanic, 3-African-American (whether Hispanic or not), 4-Asian or Pacific Islander, 5-Native American, 6-Other, more than one, or none of the above.
(PDF)

S2 Table Factor Analysis Loadings: The Two Retained Factors. (PDF)

S3 Table Impact of NFATc3 Intensity and Percent Positive Nuclei on Disease. ^1^Rho represents the correlation in the error terms across the two equations. The significance suggests that there is something unobserved that is common to both equations.
(PDF)
