# Supplementary material for: NFATc3 and VIP in Idiopathic Pulmonary Fibrosis and Chronic Obstructive Pulmonary Disease
Source: PLoS One. 2017 Jan 26;12(1):e0170606. doi: 10.1371/journal.pone.0170606 (PMC5270325; doi:10.1371/journal.pone.0170606)
Supplement: S1 Table — Standard Errors in (). Gender: 1-Male, 2-Female. Race: 1-White (Caucasian), 2-Hispanic, 3-African-American (whether Hispanic or not), 4-Asian or Pacific Islander, 5-Native American, 6-Other, more than one, or none of the above. (PDF) [file pone.0170606.s001.pdf]

S1 Table. Clinical and Demographic Data

Group	Patient	Diagnosis	Gender	Race	Age	Pkyr	FEV1	FVC	FEV1/ FVC
1	109231	COPD	1	1	78		66	81	81%
1	234573	COPD	2	1	66	40	68	81	84%
1	182880	COPD	2	1	68		102	121	84%
1	26458	COPD	1	1	73		54	63	86%
1	192758	COPD	1	1	76	3	87	100	87%
1	10491	COPD	1	1	78	51	73	82	89%
1	123457	COPD	2	1	65	35	93	104	89%
1	53341	COPD	2	1	77	27	99	109	91%
1	263091	COPD	2	1	78	110	103	101	102%
1	260111	COPD	2	1	61	50	84	80	105%
1	257433	COPD	1	1	64		101	96	105%
1	119716	COPD	1	1	59	58	70	65	108%
1	59224	COPD	1	1	77	30	113	98	115%
1	280282	COPD	1	1	80	8	68	52	131%
			42%	100%	71(2)	41(9)	84(5)	88(5)	97(4)%
2	79013	COPD	2	1	46	20	23	43	53%
2	126327	COPD	1	1	81	5	73	121	60%
2	155982	COPD	2	1	48	28	20	32	63%
2	141827	COPD	2	1	68	43	51	77	66%
2	113813	COPD	1	1	82		53	75	71%
2	294945	COPD	2	1	52	1	15	21	71%
2	20278	COPD	1	1	62	40	84	114	74%
2	231942	COPD	1	1	63	60	68	88	77%
			50%	100%	63(5)	28(8)	48(9)	71(13)	67(3)%
3	230267	COPD	2	1	55	20	13	53	25%
3	192361	COPD	2	1	50	70	12	44	27%
3	24952	COPD	2	1	61	15	16	47	34%
3	33858	COPD	2	3	53	13	28	77	36%
3	182660	COPD	1	1	58	20	17	44	39%
3	262496	COPD	2	1	59	50	16	35	46%
3	219183	COPD	1	1	45	20	17	35	49%
			71%	86%	54(2)	30(8)	17(2)	48(5)	36(3)%
4	159825	IPF	1	1	45		22	43	51%
4	264518	IPF	1	1	54	20	33	36	92%
4	1600	IPF	1	1	54	20	40	39	103%
4	104717	IPF	1	1	37	4	39	37	105%
4	111701	IPF	2	1	28		26	24	108%
4	63974	IPF	2	1	58	24	42	38	111%
4	188524	IPF	2	3	45		31	28	111%
4	219896	IPF	1	1	53	0	54	48	113%
4	67836	IPF	1	1	69		42	37	114%
4	174813	IPF	2	1	56		44	37	119%

			40%	90%	50(4)	14(5)	37(3)	37(2)	103(6)%
