# Supplementary material for: NFATc3 and VIP in Idiopathic Pulmonary Fibrosis and Chronic Obstructive Pulmonary Disease
Source: PLoS One. 2017 Jan 26;12(1):e0170606. doi: 10.1371/journal.pone.0170606 (PMC5270325; doi:10.1371/journal.pone.0170606)
Supplement: S2 Table — (PDF) [file pone.0170606.s002.pdf]

S2 Table. Factor Analysis Loadings: The Two Retained Factors

	Factor 2	Factor 2	Uniqueness
PASMC NFATc3 Intensity	0.8863	-0.0323	0.2134
PAEC NFATc3 Intensity	0.8818	-0.0987	0.2126
ASMC NFATc3 Intensity	0.0118	0.6983	0.5123
AEPC NFATc3 Intensity	0.1573	0.6833	0.5083
