# Supplementary material for: NFATc3 and VIP in Idiopathic Pulmonary Fibrosis and Chronic Obstructive Pulmonary Disease
Source: PLoS One. 2017 Jan 26;12(1):e0170606. doi: 10.1371/journal.pone.0170606 (PMC5270325; doi:10.1371/journal.pone.0170606)
Supplement: S3 Table — 1Rho represents the correlation in the error terms across the two equations. The significance suggests that there is something unobserved that is common to both equations. (PDF) [file pone.0170606.s003.pdf]

S3 Table. Impact of NFATc3 Intensity and Percent Positive Nuclei on Disease

	**IPF**	**COPD3**
**NFATc3 Intensity Indicators**		
PASMC	0.05 (0.03)	-0.08 (.03)**
PAEC	-0.02 (0.01)*	0.02 (0.01)**
ASMC	-0.02 (0.02)	0.02 (0.02)
AEPC	0.02 (0.04)	0.05 (0.04)
**% NFATc3 Positive Nuclei**		
PASMC	0.01 (0.01)	0.02 (0.01)
PAEC	-0.02 (0.01)*	0.03 (0.01)
ASMC	-0.001 (0.01)	-0.02 (0.01)*
AEPC	-0.002 (0.01)	0.01 (0.01)
**FC NFATc3 mRNA**	0.09 (0.07)	0.16 (0.07)**
OLD	-0.17 (0.18)	-0.44 (0.18)**
Rho[1]	-0.68 (0.22)**	-0.68 (0.22)**
Constant	-2.05 (1.78)	2.32 (1.78)
R-Square	0.7028	0.7028
